# Cheoplastic Dentistry

**Published:** 1857-10

**Authors:** J. M. Shepherd

**Affiliations:** Petersburg, Va.


### ARTICLE IV.

*Cheoplastic Dentistry.* By J. M. SHEPHERD, M. D., D. D. S., Petersburg, Va.

GREAT and various have been the improvements in mechanical dentistry in the last twenty years, and no one of them has created or excited a more lively interest among the dental profession than that which heads this article. The strongest emotion excited seems to be that of surprise.

It is somewhat amusing to see the laborious manipulator in gold, straighten himself up at his bench to look at a piece of Cheoplastic work. He lays down his hammer and takes up the piece, and his first inspection is made of the surface intended to fit the mouth. He finds here a perfect counterpart of the mouth—all the lines sharply drawn both in elevations and depressions. Here, says he, is a result I have not been able to reach by swaging, although I have labored at it for years. He then turns the piece over to examine the other side; and here he observes how beautifully the metal lies round the necks of the teeth; and how uniform and smooth the entire surface is without having had a tool upon it. He readily perceives that a beautiful finish can be obtained with a small amount of labor and time—more beautiful than it is possible to get in gold. He gives the plate a twist to ascertain its stiffness, and though thin, he finds that it does not spring. "That's strong enough," says he. He then takes hold of the teeth and gives them a shake, in order to test their attachment to the metal, and here he finds the new process is as far superior to his work in gold as in any other point in which he has drawn a comparison. Push them as he will they do not stir. On looking into the reason of this, he finds that the metal being cast to the tooth, instead of grinding the tooth to fit the metal, the fit is perfect; no crevice, not the least, is to be found between the two, and, consequently, the tooth sets steadily on its base; that it is confined to its place by the same platina pin which he uses, or a groove in the porcelain into which the metal has flown, and in addition, there is a neat little strip of the same metal passing round and over the ends of the teeth where they terminate on the gum in a small groove made for the purpose, and which binds them with perfect security; a thing he has tried to do in gold a hundred times, but never succeeded to any valuable extent. He concludes on the whole, that the teeth are more securely bound to the metal and more perfectly adapted to it than can possibly be done in gold. He further notices that no place is left for foreign deposits—that the piece may be worn any length of time, and when taken from the mouth, may be brushed clean in half a minute. This, though very desirable, is impossible in gold work. On further inspection he perceives that there are all necessary facilities for filling out gaunt places in the alveoli, and that a regular and uniform set of teeth may be adapted to the most irregularly shaped gum. And then he remembers the trouble and vexation he has had in the springing of his plates; nothing of the sort in the nature of things can occur here; exactly as his model, so is his plate. And, further, it is manifest at a glance, that from the nature of the metal and the manner in which it is self-braced, that it is scarcely possible by use or accident to warp the plate at any future time.

After this close examination, he lays down the piece and seems to want time to reflect. There is evidently a conflict in his mind, and he is not prepared to decide on the merits of the new process. He has already acknowledged that every essential point in the mechanical construction of a set of teeth has been achieved in a manner far beyond any process he has seen before. "But," says he, "can this metal, be worn in the mouth with impunity. He reads the certificates of Dr. Bond and others, and finds his question answered in the affirmative. Now, he wishes to reflect again. His thoughts run thus: "If this metal is pure enough to be worn in the mouth, truly it is a marvellous discovery; and, this is strong testimony on that point. Dr. Bond says he has worn plates of gold, silver and platina, and that this is as pure as any of them. But suppose this new process is adopted, what will be the result? why, it is so simple that any one can do it, and the distinction which now exists between the skillful manipulator and the botch will be done away, and we shall all be brought down on a level. Why should I abandon that which has cost me years of patient toil, and take position beside the man who never possessed industry or skill enough to put up a passable set of teeth?"

I have thus stated the case, as I think fairly, and will now present some facts in my own experience.

For more than twenty years I have been engaged in the practice of dentistry in both branches. I have always done my own artificial work, having never been able to get any one to do it for me to my mind. And I have always felt the want of some process by which greater perfection or a nearer approach to it could be attained. The double duty of the office and the laboratory, however, left me no time for experimenting with or inventing improvements. Improvements were frequently announced, however, and I adopted them, generally, without hesitation, if they appeared to be improvements. When Dr. John Allen's continuous gum came out, I was delighted with it, thinking from its looks it would stand the test; and if so, I saw that nearly all the points to which I have above referred, were most happlily met. I soon found, however, that the material of which his gum was made, was too frail and would break, even when laid on so heavily as to render the piece objectionably clumsy. About this time, some one proposed block tin as a base for lower sets, and Dr. Hawes, of New York, published his method of constructing them. I adopted this, and so much was I pleased with it, that I have never put up lower sets in anything else until the Cheoplastic process was developed by Dr. Blandy, of Baltimore. I had only one objection to block tin, and that was, it had not sufficient stiffness to admit of the use of gum teeth, and while the metallic gum could be hid in most mouths, in some it could not. But it made a more serviceable, easy and perfect base than anything else I had ever tried. But when Dr. Blandy called on me last April and showed me his new process, and specimens of work, I saw at once that he had a metal free from the objection to block tin. I thought it at least as pure, and it could be used in the construction of upper sets as well as lower, and thus obviate the necessity to use two or more metals in the same mouth.

I did not hesitate, under these circumstances, to purchase an office right. Since that time, I have put in some dozen pieces, most of them being whole dentures, and, so far as my patients are concerned, I have not heard the first word of complaint, and not a piece has come back to be repaired. Except in tin base work, I have never enjoyed so perfect an exemption from trouble with any style of work I have ever done. I am, of course, therefore, highly pleased with the Cheoplastic process.

The objections which lie against the new process, I think, may all be answered with sound argument.

That the careless and unskillful will calculate to equal his former rival in gold work, is probable; but, when he comes to make a comparison with well finished pieces, he will find himself vastly mistaken. I do not recommend it on the score of its simplicity; my experience with it is, that more skill, if possible, is requisite to compass all the ends practicable in this style of work than in gold. The effect of nicety is not seen until the piece is cast, and then if defective, it must remain so. The most perfect nicety is requisite in fitting and arranging, in order to get out a handsome piece, or a piece that can be made handsome by finishing. But there is no style of work with which I am acquainted that responds so amply to the skill of the manipulator as does this. He fails in nothing for which he labors; his highest aims are fully and perfectly met. But it requires skill of the most superior order to bring out the best results.

It is probable that the greatest difficulty on the minds of the profession in reference to Cheoplasty, will be the metal itself. The quality of metal for all dental purposes has rightly claimed the attention of all well instructed dentists. Tin foil was extensively used in filling teeth twenty years ago, and from the fact that it was found to corrode in some localities of some mouths, it was discarded from the operating cases of most of the profession. But it must be borne in mind that there is a wide difference between a filling in a tooth and a piece of the metal cast in a solid mass. The filling moreover, is stationary in the mouth and may be neglected, while artificial teeth are removed and cleansed at pleasure. It is known to all who have been many years in practice that a filling of tin foil will last indefinitely in a healthy mouth, and preserve the tooth from decay, too, if kept clean. I have at least two tin fillings in my own teeth that were put in by my dental preceptor twenty-two years ago; and the teeth are perfectly preserved, although they were in an advanced stage of decay when they were filled. The patentee has wisely withheld from his patrons the formula by which his metal is compounded; and, I think the reason which he assigns is satisfactory—that the metal would almost certainly degenerate if indiscriminately compounded. But I am willing to believe that it is at least as pure as block tin; and if so, time has already proved to me that it may be worn in the mouth with impunity.

There is, however, a charm about gold which it is hard to dispel; and I confess if gold could be cast, or wrought by any other means so as to meet the requirements as this new metal does I would use it in preference, not because I think it would be intrinsically better, but because there would be no objections to combat in the use of it. In order to meet this fancy for gold, so far, I have gilded all my pieces. It may be an intrinsic advantage—I am sure it does no harm, and it makes a piece look better, and my patients like it better. Gilding is a simple process, and may be done by any dentist in his own laboratory with a small outlay of time and money.

Some difficulty has been experienced in the want of a proper style of teeth for the new process. While the ordinary teeth may be used with facility, they do not fulfil all the requirements. Several styles have been brought out, but I have succeeded best with a tooth made by my own model, by Messrs. Orum and Armstrong, of Philadelphia. They are, or soon will be, prepared to fill orders. No doubt there will soon be in the market a good supply of various styles to suit all fancies.

There is one objection in the minds of many excellent men in the profession, to which I have not referred; they are opposed to professional patents. Well, so am I as a principle. But I have always adopted the improvements without stopping to discuss the question of propriety in relation to such patents. The improvements are essential to me, and if I cannot get them without paying the fee of the patentee, I pay the fee and discuss the other question afterwards.

I have thus written in order to direct the attention of my professional brethren, if possible, more directly to this, as I esteem it, great improvement in artificial dentistry. I am so well satisfied that it is not one of those discoveries which only attract to deceive, but will stand the test of time, and become the prevailing mode of mounting teeth, that I am willing to give it my sanction before the profession as well as in my laboratory.

